# ﻿A new species of *Monstrilla* (Copepoda, Monstrilloida) from the western Caribbean with comments on *M.wandelii* Stephensen, 1913 and *M.conjunctiva* Giesbrecht, 1893

**DOI:** 10.3897/zookeys.1128.84944

**Published:** 2022-11-04

**Authors:** Eduardo Suárez-Morales

**Affiliations:** 1 El Colegio de la Frontera Sur (ECOSUR), Unidad Chetumal, 77014 Chetumal, Mexico Unidad Chetumal Chetumal Mexico

**Keywords:** Mexican Caribbean, Monstrillidae, morphology, protelean endoparasites, taxonomy

## Abstract

The taxonomic study of monstrilloid copepods is hampered by incomplete early descriptions, uncertain synonymies, and the difficulty of reliably matching males and females of species. A re-evaluation of male monstrilloid specimens collected from two reef areas of the Mexican Caribbean allowed me to clarify the status of *Monstrillamariaeugeniae* Suárez-Morales & Islas-Landeros, 1993 and *M.wandelii* Stephensen, 1913 based on a comparison of males attributed to each of these species. Males from the Puerto Morelos reef system, northern Mexican Caribbean coast, were first proposed as a tropical subspecies of the subarctic *M.wandelii*; later on, morphologically close males collected from the Mahahual reef area, southern Mexican Caribbean coast, were designated as the males of *M.mariaeugeniae*. Their status is here corrected with the description of *M.mahahualensis***sp. nov.** based on the Mahahual males; the new species shares the same type of genitalia with the antarctic *M.conjunctiva* Giesbrecht, 1892 and the subarctic *M.wandelii*; Park (1967) linked a single male from Vancouver to *M.wandelii*. It was realised that Park’s (1967) males from the Vancouver area and the two Mexican Caribbean groups of males represent different, undescribed species. I here reassign the males earlier attributed to *M.mariaeugeniae* as a new species of *Monstrilla* which is herein described. The new species differs from the males of *M.conjunctiva* and *M.wandelii* by details of the genitalia, length of the setae of the fifth legs, armature and integumental structures of the antennules, and size of the outer exopodal spines of legs 1–4. This is the third known species of *Monstrilla* with a *M.conjunctiva*-like male genitalia and the first one known from tropical areas. The male of both *M.mariaeugeniae* and *M.wandelii* remain unknown.

## ﻿Introduction

Monstrilloid copepods are protelean endoparasites infecting different groups of marine invertebrates including polychaetes, molluscs, and sponges (Huys et al. 2007; Suárez-Morales 2011, 2018; Jeon et al. 2018) and are frequently recorded as adults in plankton samples. Their taxonomic and nomenclatural history is complex and uncertain (Grygier and Ohtsuka 2008; Suárez-Morales 2011, 2018; Suárez-Morales and Grygier 2021), particularly in reference to old records, early species descriptions, and the recognition and matching of males and females of the species (Grygier 1994, 1995; Suárez-Morales 2010, 2011, 2018; Suárez-Morales and Grygier 2021). The re-examination of collection specimens and the analysis of material from new sites with updated taxonomic criteria are valuable tools to solve some of these taxonomic problems (Grygier 1994; Suárez-Morales 2000a, b; Suárez-Morales and Gasca 2004).

## ﻿Materials and methods

During the examination of zooplankton samples from Mahahual reef, southern coast of the Mexican Caribbean, several male monstrilloids were re-examined. They were tentatively identified by Suárez-Morales (1998) as the males of *M.mariaeugeniae* Suárez-Morales & Islas-Landeros, 1993, originally described from females collected at Puerto Morelos (PM) reef, on the northern coast of the Mexican Caribbean. Suárez-Morales (1996) designated PM males as a tropical subspecies of the Arctic *M.wandelii* Stephensen, 1913.

In order to determine the status of the northern and southern Mexican Caribbean males, which was still uncertain, I re-examined the Mahahual male specimens. I sorted five adult males closely resembling those that were previously named by me (Suárez-Morales 1996) as a subspecies (i.e., *Monstrillawandeliitropica*) from the northern Mexican Caribbean reef (PM) and subsequently designated as the males of *M.mariaeugeniae* (Suárez-Morales 1998) to determine their status in the light of their resemblance to males from PM and to other species. As an outcome of this new evaluation using the descriptive criteria by Grygier and Ohtsuka (1995), the male individuals from Mahahual are here recognized as a new species, *M.mahahualensis* sp. nov., and not a *M.wandelii* subspecies nor the males of *M.mariaeugeniae*. The new species from Mahahual is fully described including examination with SEM and compared with similar species and male specimens from PM. The female of the new species remains unknown, like the true males of *M.mariaeugeniae*. Also, the male of *M.wandelii* from Vancouver, as described by Park (1967), is likely to represent an undescribed species; the male of *M.wandelii* from Greenland remains unknown.

## ﻿Results

### ﻿Taxonomy

**Subclass Copepoda Milne Edwards, 1840**

**Order Monstrilloida Sars, 1901**

**Family Monstrillidae Dana, 1849**

#### Genus *Monstrilla* Dana, 1849

##### ﻿ Monstrilla mahahualensis sp. nov.

Taxon classificationAnimaliaMonstrilloidaMonstrillidae

ABB19FFC-AA12-5340-BD25-0B43032845EF

https://zoobank.org/4761E010-CB8C-4F53-AC2B-F94E18AA6E16

Figs 1 , 2 , 3 , 4

###### Type material.

Adult male ***holotype***, partially dissected (ECO-CH-Z 10595); specimen mounted in glycerine, sealed with acrylic varnish; two slides, collected by L. Vásquez-Yeomans and A. González-Vera, December 31 1990, plankton sample; ***Paratypes***: 3 adult males (ECO-CH-Z 10596), one mounted on slide, undissected, and 2 males in vial, ethanol-preserved, undissected; collection data as holotype. Additional non-type material: one adult male prepared for SEM examination following the procedures described by Silva-Briano et al. (2013).

###### Type locality.

Reef lagoon of Mahahual (18°43'11.42"N, 87°42'11.01"W), southern coast of the Mexican Caribbean.

###### Etymology.

The species epithet, a toponym in singular, refers to the reef system of Mahahual, the type locality of this species. The gender is feminine.

###### Diagnosis.

Male *Monstrilla* with light cuticular reticulation of cephalothorax covering cephalic area and 2/3 of post-oral cephalothorax surface. Antennule 5-segmented, geniculate, segments 3 and 4 indistinctly segmented, intersegmental division marked by constriction. First antennulary segment unarmed, segments 3 and 4 each with discoid integumental structures of unknown function; armature of segments 4 and 5 reduced. Element 4d1 (Grygier and Ohtsuka 1995) robust, spinulate. Legs 1–4 with relatively long outer exopodal spines. Genital complex type II (Suárez-Morales 2000c) with short, thick shaft carrying pair of lappets subdistally. Lappets simple, conical, weakly asymmetrical, with outer surface furnished with rows of spinules. Fifth legs represented by pair of bulbous processes armed with long distal seta reaching beyond posterior margin of caudal rami.

###### Description of adult male.

**Figure 1.**
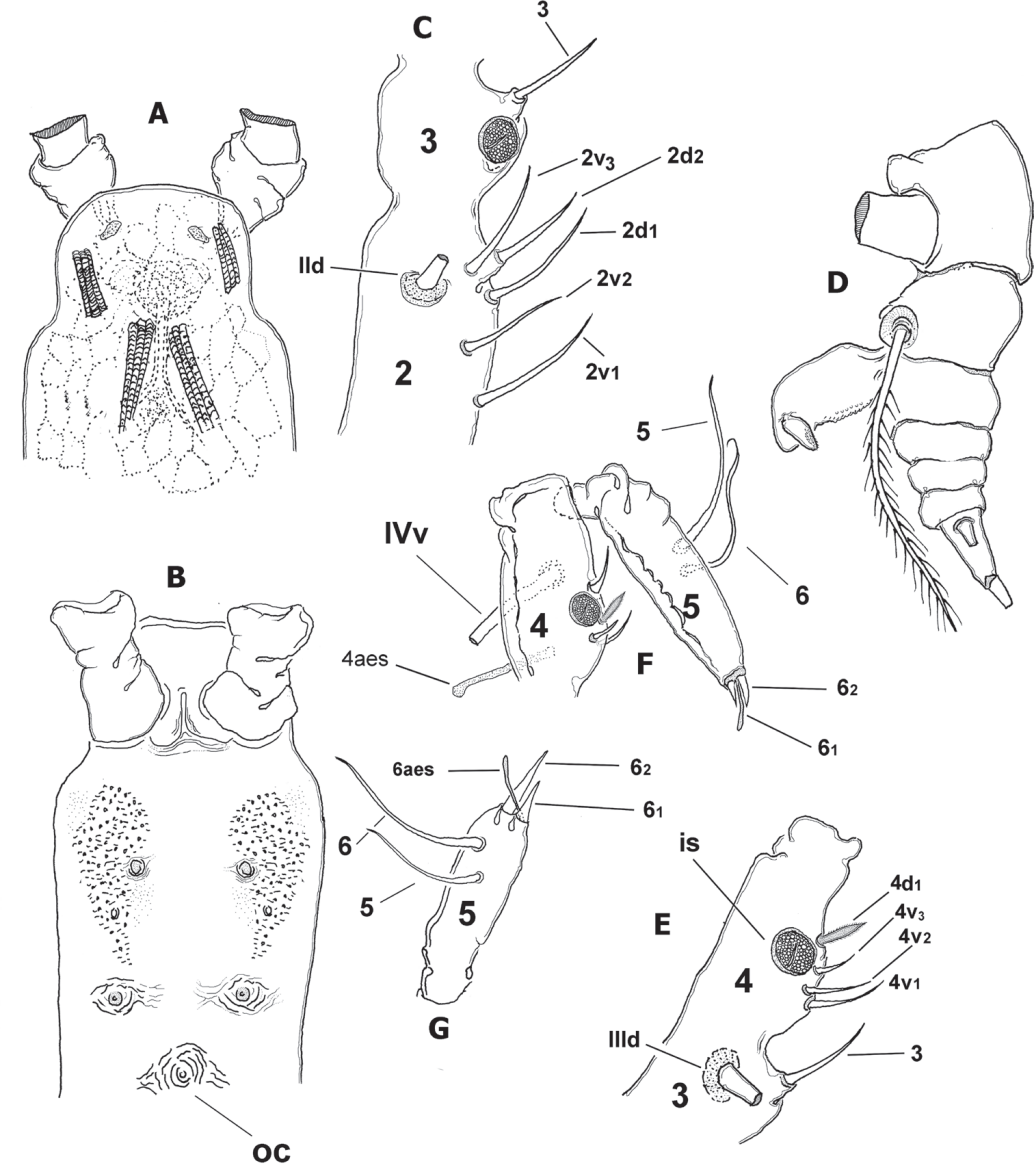
*Monstrillamahahualensis* sp. nov., holotype adult male from Mahahual, Mexico **A** cephalic region showing weak reticulation pattern, dorsal view **B** perioral area showing oral cone (oc), cuticular processes and ornamentation, ventral view **C** antennule segments 2 and 3 showing setation pattern and position of integumental structure **D** urosome showing fifth legs and genital complex, lateral view **E** third and fourth antennule segments showing setation pattern and position of integumental structure (is) **F** fourth and fifth antennule segments showing setation pattern and position of integumental structure **G** fifth antennule segment showing setation pattern Scale bars: 50 μm (**A–G**).

**Figure 2.**
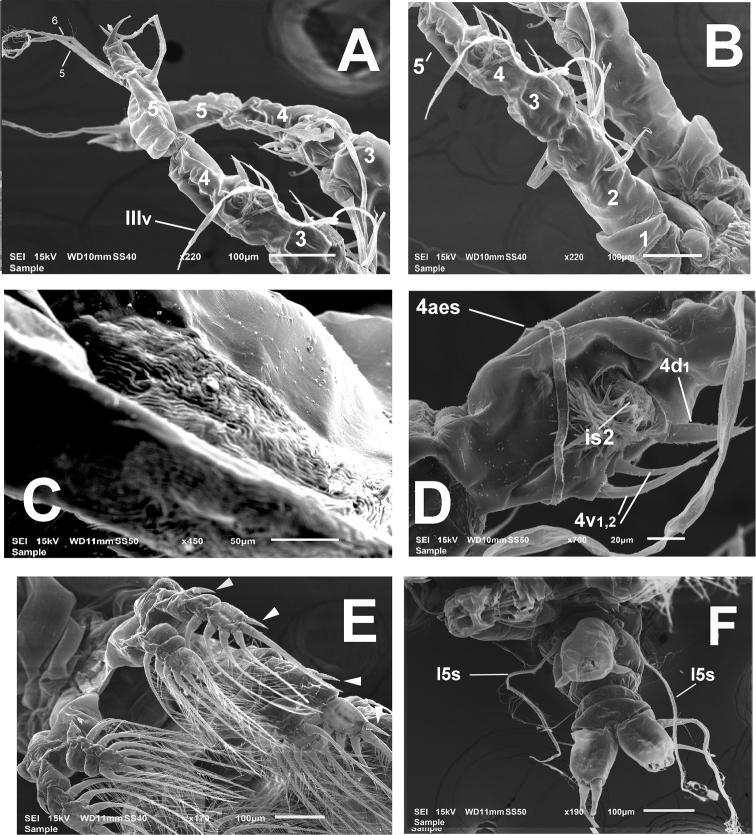
*Monstrillamahahualensis* sp. nov., SEM-prepared adult male from Mahahual, Mexico **A** antennules, ventral view indicating purported segments 3–5 **B** same, semi- lateral view indicating purported segments 1–5 **C** detail of wrinkled cuticular field on perioral surface, ventral view **D** purported fourth antennulary segment showing setation pattern and second integumental structure (is2) **E** legs 1–3 showing endopodal and exopodal rami general setation pattern, arrows indicate relatively long outer exopodal spines, ventral view **F** urosome with fifth legs armed with long setae, and genital complex, ventral view. Setal elements on antennule segments 1–4 labeled following Grygier and Ohtsuka’s (1995) nomenclature.

Body size of holotype 2.56 mm, of one paratype 2.72 mm measured from forehead margin to posterior end of anal somite. Body tagmosis as usual in males of *Monstrilla* (Suárez-Morales and Castellanos-Osorio 2019; Suárez-Morales 2000c; Suárez-Morales 2022). First pedigerous thoracic somite incorporated into cephalothorax. Cephalothorax long, cylindrical, relatively robust, representing about 60% of total body length. Oral cone located at 40% of way back along ventral surface of cephalothorax (Fig. 1B). Cephalic region anteriorly subquadrate, forehead flat (Fig. 1A). Ocelli poorly developed, almost unpigmented. Small oval hyaline bodies (see Suárez-Morales 2018; Fig. 1A) adjacent to rounded ocelli. Cuticular ornamentation of cephalothorax including light cuticular reticulation showing irregular pattern covering cephalic area and almost 2/3 of post-oral cephalothoracic surface (Fig. 1A). On ventral surface, three pairs of nipple-like processes present between oral cone and antennule bases (Fig. 1B); two foremost ventral processes with adjacent pattern of minute transverse wrinkles and small papillae (Figs 1B, 2C). Oral cone moderately produced, with adjacent field of faint, transverse striae (oc in Fig. 1B).

**Figure 3.**
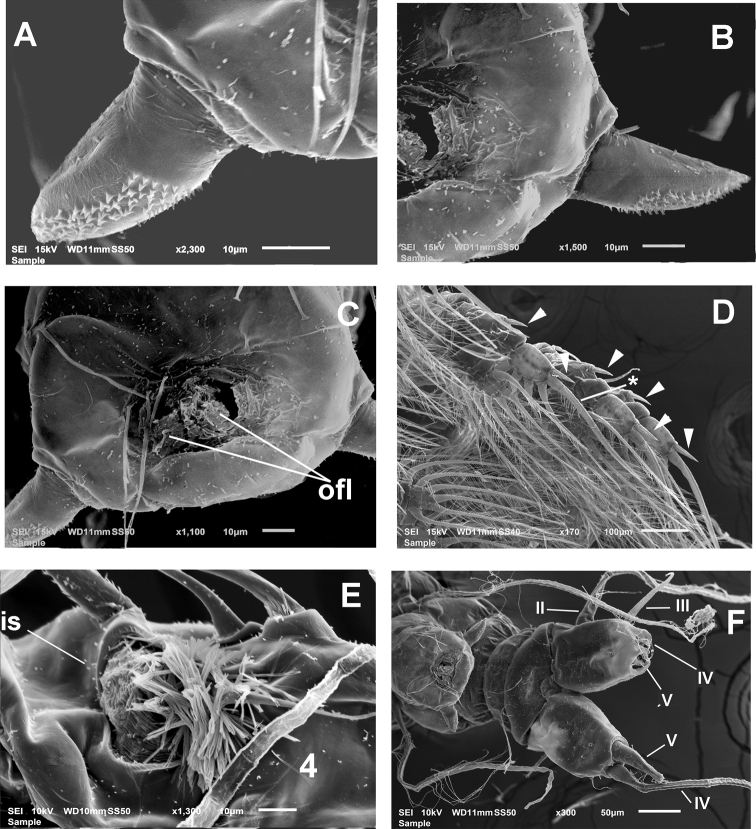
*Monstrillamahahualensis* sp. nov., SEM-prepared adult male from Mahahual, Mexico, female holotype **A** detail of right genital lappet showing cuticle ornamentation, ventral view **B** same, left genital lappet, semi-lateral view **C** apical surface of genital complex showing genital opening and paired opercular flaps (ofl), ventral view **D** swimming les 2–4 showing setation pattern and long exopodal spines (arrowheads) **E** integumental structure on antennule segment 4, ventral view **F** urosome, genital complex, and caudal rami showing setation pattern (setae II–V).

Urosome consisting of five somites: fifth pedigerous somite (largest of urosome, with fifth legs), genital somite (with genital complex on ventral surface), two free somites, and short anal somite. Length ratio of urosomites (from proximal to distal) being: 44.1: 24.4: 12.6: 10.1: 8.8 = 100 (Figs 1D, 2F, 3F).

**Figure 4.**
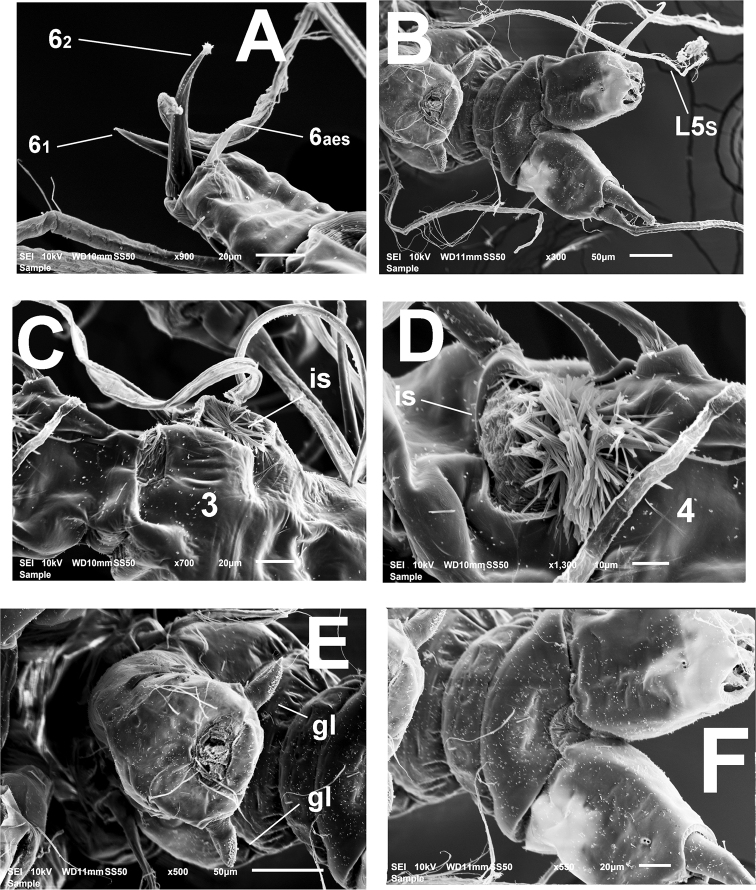
*Monstrillamahahualensis* sp. nov., SEM-prepared adult male from Mahahual, Mexico **A** right antennule fifth segment showing terminal setal elements, ventral view **B** distal part of urosome showing genital complex, fifth legs with long setae (L5s) **C** integumental structure 1 (*is*), on third antennulary segment, semi-lateral view **D** integumental structure 2 (*is*) on fourth antennulary segment, ventral view **E** genital complex showing genital opening and paired genital lappets(*gl*) **F** detail of anal somite, ventral view. Setal elements on segments 1–4 labeled following Grygier and Ohtsuka’s (1995) nomenclature; armature of fifth segment labeled following Huys et al. (2007); ae2 on distal segment follows Huys et al. (2007) sequence notation of antennulary aesthetascs. Arrows indicate proximal rounded protuberance on fifth antennulary segment. Scale bars: 75 μm (**A, B**).

Antennules representing about 40% of total body length, and almost 65% of cephalothorax length. As usual in males of *Monstrilla*, antennules indistinctly five-segmented, geniculation between segments 4 and 5 (Figs 1F, 2A, B). Following setal nomenclature proposed by Grygier and Ohtsuka (1995) for female monstrilloid copepod antennules. First segment lacking setal element 1. Second segment carrying six elements, including short elements 2v_1-3_, and 2d_1,2_ plus long seta IId, with broad socket (Fig. 1C). Second and third segments separate. Third segment short, globose, with setiform flexible medial element 3 reaching about proximal 1/3 of fourth segment; inner surface of segment with discoid integumental structure of unknown function (Fig. 1C, *is* in Figs 1E, 3E, 4C, D) at insertion of element 3; structure present in the right antennule only. Setae IIId, IIIv absent. Fourth segment fused with third, division marked by moderate constriction; segment armed with five setal elements: 4d1, 4v_1-3_, IVv, and long aesthetasc (4aes) (Fig. 1F, 4D); discoid integumental structure like that on third segment present at insertion of elements 4d_1_ and 4v_1-3_ (Figs 1E, 2D, 3E). Setal element 4v_1_ longer than 4v2, 3; element 4d1 robust, spiniform, pinnate (Fig. 1E). Distal fifth segment geniculate, slightly longer than preceding fourth segment (Fig. 1F), with reduced armature, armed with six setal elements (*sensu*Huys et al. 2007) (see Table 2). Spiniform apical elements 61 and 62 (*sensu*Grygier and Ohtsuka 1995), or 1,2 (*sensu*Huys et al. 2007) present, unequally long (Figs 2A, 4A). Length ratio of antennular segments (proximal to distal): 11.4: 19.8: 11.4: 27.8: 29.6 = 100 (Fig. 2A, B).

First incorporated pedigerous thoracic somite and succeeding three thoracic somites each bearing well-developed biramous swimming legs (Figs 2E, 3D). Swimming legs 1–4 as in *M.mariaeugeniae* (Suárez-Morales and Islas-Landeros 1993, fig. 1c, d, i, j), all with triarticulate endopodites and exopodites and same armament pattern except for leg 1 exopodite, bearing one seta fewer on distal segment (Fig. 1E). Exopodites longer than endopodites. Distal spiniform seta of third exopodite with denticles along outer margin, inner margin weakly setulose in all legs (* in Fig. 3D). Outer spines of exopodal segments 1 and 3 of all legs noticeably long (arrowheads in Fig. 3D). Basis of swimming legs 2–4 with short basipodal seta; basipodal seta on legs 1 and 2 not observed; seta on leg 3 long. All natatory setae lightly and biserially plumose. Huys and Boxshall (1991) was followed for general and setation nomenclature. Armament formula of legs1–4 as in Table 1.

**Table 1.** Armature of swimming legs 1–4 including basipodites, exopodites and endopodites. Roman numerals indicate spiniform elements, Arabic numbers indicate setiform elements.

	Basipodite	Endopodite	Exopodite
Leg 1	1-0	I-1;0-1;,2.2.1	I-0;0-1; I,2,2
Legs 2–4	1-0	0-1;0-1;2,2,1	I-1;0-1; I,2,2,1

Fifth legs reduced, represented by pair of small globose protuberances on ventral surface of fifth pedigerous somite armed with long single seta reaching beyond posterior margin of caudal rami (Figs 1D, *l5s* in Figs 2F, 3F, 4B). Genital somite carrying genital complex consisting of short, thick medially curved shaft bearing paired subdistal genital lappets (Figs 2F, 3A–C, 4B, E). Lappets divergent, conical, tapering distally, ornamented with rows of spinules on inner surface (Fig. 3A, B); genital complex carrying genital opening with pair of rounded opercular flaps in apical position (*ofl* in Fig. 3C).

Caudal rami subrectangular, each ramus approximately 1.3 times as long as wide, bearing four caudal setae (setae II–V; Huys and Boxshall 1991), armature incomplete in some specimens, but sockets indicate setal insertions (Figs 2F, 3F, 4B).

**Female.** Unknown.

## ﻿Discussion

The male specimens studied here can be included in the genus *Monstrilla* by 1) the presence of two urosomites between the genital somite and the anal somite, 2) the structure of its genital complex that is typical for the genus, in this case, type II (Suárez-Morales 2000d); 3) the presence of well-developed or reduced paired fifth legs, 4) the possession of 4–6 caudal setae in both sexes, and 5) branched antennulary setae sometimes present. This combination of characters is unique among the known genera of monstrilloids (Grygier and Ohtsuka 2008; Suárez-Morales and McKinnon 2014; Jeon et al. 2018).

The males of *Monstrillaconjunctiva* Giesbrecht, 1902, *M.wandeliisensu*Park (1967), and *M.mahahualensis* sp. nov., share a similar structure of the genitalia. This type of male genital complex was first described and depicted for *M.conjunctiva* by W. Giesbrecht from a male collected in Antarctic waters (Giesbrecht 1902). There are no other known species of *Monstrilla* with the *M.conjunctiva*-like male genitalia; therefore, the comparisons in this work include the three nominal species of monstrilloids sharing this character (Table 2).The shape, structure, and ornamentation of the genital lappets, the length of the fifth leg setae, body size, and antennule setation pattern show some differences among these three taxa, as follows: the genital lappets are simple, leaf-like, distally acute in *M.conjunctiva* (Giesbrecht 1902, fig. 12.3) whereas they are chelate and smooth in *M.wandelii* (see Park 1967, fig. 2C, D); in *M.mahahualensis* sp. nov., the lappets are simple, conical, and ornamented with rows of spinules (Fig. 3A, B). Also, in both *M.conjunctiva* (Giesbrecht 1902, figs XII.3, 4) and *M.wandeliisensu*Park (1967, fig. 2C, D), the leg 5 setae are relatively short, not reaching the distal margin of the caudal rami. In the new species, *M.mahahualensis*, the fifth leg setae reach well beyond the posterior margin of the caudal rami (Figs 2F, 3F, 4B). The new species shares with *M.wandeliisensu*Park (1967) the relatively long outer exopodal spines of legs 1–4, and the antennule segmentation and armature pattern (see Table 2). It shares with *M.conjunctiva* the caudal rami setation pattern and the antennule setation (Table 2).

**Table 2.** Comparison of male antennule armature patterns of different species and populations with *M.conjunctiva*-like genitalia. Identification of elements on A1 segments 1–4 followed Grygier and Ohtsuka’s (1995) nomenclature; for elements on A1 segment 5, Huys et al.’s (2007) nomenclature was followed.

A1 elements (*sensu*Grygier and Ohtsuka 1995)	*M.mahahualensis* sp. nov.	*M.conjunctiva* Giesbrecht, 1902	*M.wandelii* Park (1967)
61/62 ratio	0.66	0.4	0.9
Element 1	absent	absent	absent
Elements on S2	2v_1-3_, 2d_1,2_, IId	2v_1-3_, 2d_1,2_	2v_1-3_, 2d_1,2_, IId
Elements on S3	IIIv, IIId, 3	IIIv, IIId, 3	IIIv, IIId, 3
Elements on S4	4v_1-3_, 4d_1,_ 4aes, IVv	4v_1-3_, 4d_1,_ 4aes, IVd	4v_1-3_, 4d_1,2_, 4aes, IVv
Elements on S5*	1,2,6, C,B AE2	1, 2, 4, 6, C, AE2	1, 2, 4, 6,C, AE2

The rounded integumental structures resembling those here observed in *M.mahahualensis* sp. nov. were first depicted, but not described, by Suárez-Morales (1996, fig. 2A, B) in males of PM. These structures were not reported by Giesbrecht (1902) or Park (1967) and are present in the female holotype of *M.mariaeugeniae* (pers. obs.), thus agreeing with Suárez-Morales’ (1996) notion that the PM males, and the Mahahual males (Suárez-Morales 1998) were linked to *M.mariaeugeniae*.

There are two distinct types of male genitalia among species of *Monstrilla* and of the related genus *Caromiobenella* Jeon, Lee & Soh, 2018 (McAlice 1985; Jeon et al. 2018; Suárez-Morales 2000d; Cruz Lopes da Rosa et al. 2021): the first one (type I) shows a genital shaft of variable length and thickness bearing a deep distal notch between the distally set lappets; this medial notch is frequently ornamented with spinules or teeth on its inner surface. A second type (type II) is distinguished by the presence of a smooth, rounded medial protrusion instead of a notch in the same position. The male genitalia displayed by the three species: *M.wandeliisensu*Park (1967), *M.conjunctiva*, and *M.mahahualensis* sp. nov. are assignable as a modified type II male genitalia, in which lappets are widely divergent, probably resulting from a strong development of the broad medial protrusion between them.

The antennule segmentation, with fused segments 3 and 4 is another character shared by the three species, *M.wandelii*, *M.conjunctiva*, and *M.mahahualensis* sp. nov., including their reduced setation of segments 1, 4, and 5. The setation patterns of segments 1–4 were compared following Grygier and Ohtsuka’s (1995) nomenclature for the first time. The differences among these species and Caribbean males are presented in Table 2.

The PM males share several characters with *M.mahahualensis* sp. nov., namely: 1) antennule segmentation pattern, 2) body proportions, cephalothorax ornamentation, 3) caudal rami armature, 4) antennule armature (see Table 1), 4) position of the oral papilla (~ 40% of way back along ventral surface of cephalothorax), and 5) the presence of integumental structures on the antennule (Suárez-Morales 1996, fig. 2A, B). There are, however, some important differences between the PM males and *M.mahahualensis* sp. nov. from Mahahual (southern Mexican Caribbean), as follows: In the PM males the fifth legs setae are relatively short, not reaching beyond the posterior margin of the caudal rami (Suárez-Morales 1996, figs1B, 2C), thus diverging from the clearly longer fifth leg setae of *M.mahahualensis* sp. nov. reaching well beyond the distal margin of the caudal rami; 2) in addition, the genital lappets of the PM males are bilobed, chela-like (Suárez-Morales 1996, figs 2C, 3A) vs. simple, conical in *M.mahahualensis* sp. nov. (Fig. 3A, B). Also, the PM males have two integumental structures on antennulary segment 2 (Suárez-Morales, 1996, fig. 2A, B), but not on segments 3 and 4, as observed in *M.mahahualensis* sp. nov. (Figs 1E, F, 2D, 3E). Antennulary segments 6_1_ and 6_2_ are equally long in the PM males (Suárez-Morales 1996, fig. 2A, B) and unequally long in *M.mahahualensis* sp. nov. (Figs 1G, 2A, 4A, Table 1). Thus, it is likely that the PM males represent a different species closely related with *M.mahahualensis* sp. nov. The holotype of the PM species is deposited in the USNM collection as *Monstrillawandeliitropica* (USNM-259668) and is probably a different species (see Suárez-Morales 1996), whose link with *M.mariaeugeniae* is yet to be determined.

## Supplementary Material

XML Treatment for Monstrilla mahahualensis
